# Management of Complicated Crown Fracture with Miniature Pulpotomy: A Case Report

**Published:** 2014-07-05

**Authors:** Saeed Asgary, Mahta Fazlyab

**Affiliations:** a Iranian Center for Endodontic Research, Research Institute of Dental Sciences, Shahid Beheshti University of Medical Sciences, Tehran, Iran; b Dental Research Center, Research Institute of Dental sciences, Shahid Beheshti University of Medical Sciences, Tehran, Iran

**Keywords:** Calcium-Enriched Mixture, CEM cement, Complicated Crown Fracture, Crown Fracture, Miniature Pulpotomy, Pulp Exposure, Vital Pulp Therapy

## Abstract

Crown fractures account for the majority of dental traumas. If handled properly, prognosis of the pulp following a traumatic crown fracture can be favorable. The present case report focuses on the treatment of a traumatized mature permanent incisor with exposed pulp that was treated with the novel technique of miniature pulpotomy using calcium-enriched mixture (CEM) cement. One-year follow-up revealed that the tooth was responsive to vitality tests and radiographic assessment showed the presence of a thick dentinal bridge beneath the CEM layer.

## Introduction

Crown fractures represent the majority of dental trauma in the permanent dentition (26–76% of dental injuries) [1]. Crown fractures fall into the following categories: *i)* Fracture of enamel of the tooth (enamel infraction), *ii)* Fracture of crown (enamel-dentin) of tooth without pulpal involvement and *iii)* Fracture of crown of tooth with pulpal involvement [2, 3]. 

The success of treatment and prognosis of the traumatized tooth depends on accurate diagnosis and treatment procedures and materials [2]. Various factors can make an impact on treatment choice/success including the time elapse between pulp exposure and treatment, health status of the pulp, diameter of exposure, patient’s age and tooth maturity and presence/absence of concomitant laxative injuries that can threaten the tooth nutrition and the pulp’s healing ability [3].

In young patients with immature teeth, preservation of the pulp vitality by various vital pulp therapy (VPT) techniques such as pulp capping or pulpotomy, is very important [4]. VPT is also the choice in mature teeth of younger patients. In older patients, root canal treatment can be the treatment of choice, although pulp capping or pulpotomy may also be selected [5].

Miniature pulpotomy was defined as a type of VPT that can substitute direct pulp capping with limited removal of infected dentin chips/damaged pulp tissue specially injured odontoblast cells that would not exceed ~1mm; this treatment modality ensures a clean surgical wound and improves the interaction of pulp covering agent with the undifferentiated mesenchymal cells [6].

The present case report will focus on miniature pulpotomy of a traumatized mature incisor with complicated crown fracture and represents the one-year successful outcomes of this treatment.

## Case Report

**Figure 1:**
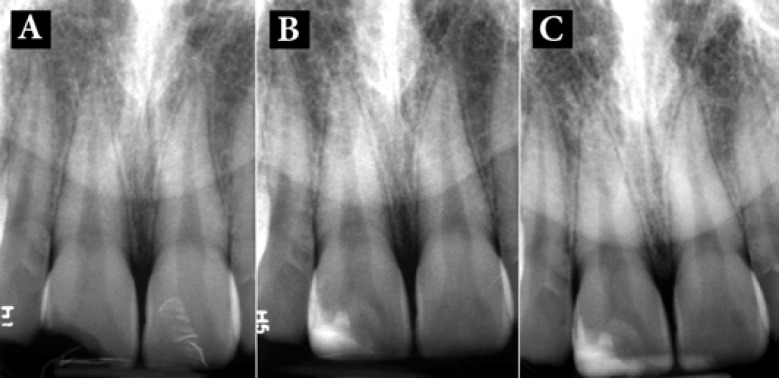
*A) *Pre-operative periapical radiography of the maxillary right incisor with complicated crown fracture and pulp involvement; *B)* Post-operative periapical radiography after miniature pulpotomy and crown restoration; *C)* One-year follow-up; the tooth was vital, functional and symptom-free; note the thick dentinal bridge beneath CEM cement

A young girl in her early twenties presented with a history of trauma to her upper right incisor two days prior to her appointment in private practice. Her chief complaint was a sharp pain with short duration during eating and drinking. Compared to the other incisors the tooth was hypersensitive to Endo-Frost cold spray (Coltene Whaledent, Langenau, Germany). The clinical appearance of the tooth showed a disto-incisal breakage that had exposed the pulp and its surrounding dentin. On a parallel radiography the tooth had a mature apex and the distal pulp-horn was evidently involved (Figure 1A).

After a mouth rinse with 0.2% Chlorhexidine gluconate (Behsa Co., Tehran, Iran), the tooth was anesthetized (2% lidocaine with 1:80000 epinephrine; Darupakhsh, Tehran, Iran) and isolated. The surface of dentin was refreshed with a #4 round diamond bur (D&Z, Wiesbaden, Germany) and the exposed surface of the pulp was gently touched with a sterile round diamond bur in presence of copious water-cooling; the total removal of the pulp did not exceed ~1 mm. After gaining hemostasis with a wet cotton pellet, CEM cement powder and liquid (BioniqueDent, Tehran, Iran) were mixed according to the manufacturer’s instructions. A small bulk of the prepared cement was gently condensed into the pulpal cavity and the rest of the tooth structure was restored with composite resin (3M ESPE, St. Paul, MN, USA). After taking a control post-op radiography (Figure 1B), the patient was dismissed and was put on a regular follow-up.

Figure 1C shows the one-year follow-up radiography and the image shows formation of a thick dentinal bridge beneath the biomaterial as well as periradicular normal contour and width of PDL. At the time of follow-up, the patient was symptomless. 

## Discussion

A less invasive form of pulpotomy, miniature pulpotomy adheres to more biologic principles and conservative hypothesis to achieve better clinical outcomes [6]. The procedure will encourage the outcomes of direct pulp capping by creating a clean surgical wound and gaining closer proximity to the biomaterial covering the pulp and pulpal stem cells [6, 7]. Moreover, miniature pulpotomy provides access to underneath layers with minor pulpal inflammation, and therefore, leading to better control of bleeding and subsequent clot free pulp wound. In addition, by creating a cavity with approximately parallel walls, it creates enough space for a suitable thickness of pulp capping biomaterial, thus leading to an optimal seal [6].

The correct choice of a pulp capping biomaterial is the main factor in success of a VPT treatment in traumatized teeth with exposed pulps; providing a tight seal by the biomaterial will prevent further bacterial contamination of the treated pulp [5]. Reviewing of scientific evidence indicates that the application of CEM biomaterial with acceptable physical seal in various VPTs leads to favorable clinical outcomes [5].

## Conclusion

Considering the favorable outcomes of miniature pulpotomy using CEM cement, this treatment may be routinely used for traumatized exposed pulps.
